# Comparison of Longitudinal Changes in Refractive Error of Hyperopic Children with or without Refractive Accommodative Esotropia

**DOI:** 10.3390/diagnostics11091547

**Published:** 2021-08-26

**Authors:** Ji Eun Song, Hyo Ji Han, Chul Young Choi, Ramin Khoramnia, Hae Ran Chang, So Young Han

**Affiliations:** 1 Department of Ophthalmology, Kangbuk Samsung Hospital, Sungkyunkwan University School of Medicine, Seoul 03181, Korea; jj.song@samsung.com (J.E.S.); hyoji.han@samsung.com (H.J.H.); sashimi0@naver.com (C.Y.C.); hrch0523@hanmail.net (H.R.C.); 2 The David J. Apple International Laboratory for Ocular Pathology and International Vision Correction Research Centre (IVCRC), Department of Ophthalmology, University of Heidelberg, 69120 Heidelberg, Germany; ramin.khoramnia@med.uni-heidelberg.de

**Keywords:** refractive accommodative esotropia, hyperopia, refractive error

## Abstract

We investigated longitudinal changes in the spherical equivalent refractive error (SE) in hyperopic children with or without refractive accommodative esotropia (AccET). A total of 456 patients met the inclusion criteria: 190 (41.7%) in the hyperopic control group and 266 (58.3%) in the AccET group. All patients received at least 3 years of follow-up after spectacle prescription. Subgroups were divided according to age when spectacles were prescribed, presence of amblyopia, or initial SE. Longitudinal changes in SE in children with hyperopia showed a gradual decrease, although SE of younger children with AccET increased over the first 4 years and then decreased thereafter. SE in eye with higher SE was tended to decrease significantly in patient with Acc ET than hyperopic control group (group × time *p* = 0.015). Amblyopic eyes showed a greater decreased in SE compared with non-amblyopic eyes, but it was not statistically significant (*p* = 0.07). SE was significantly decreased in children with more hyperopia (≥ 3 D) compared with children with less hyperopia (<3 D) (*p* = 0.008). Emmetropization of hyperopia was faster in hyperopic patients without AccET and could be affected by the age of the initial spectacles prescription, initial amount of SE, or presence of amblyopia.

## 1. Introduction

Esotropia is a disorder of ocular malalignment characterized by an inward deviation of the eyes and is diagnosed in up to 1 in 25 children [1,2]. Accommodative esotropia is a type of esotropia that occurs due to efforts of accommodation, and if eye position is fully corrected with hyperopic correction, it is called refractive accommodative esotropia (AccET). Accommodative esotropia accounts for approximately one half of the esotropia patients in United States [1,3]; however, the incidence rate of esotropia in Asians is lower than that of Western populations [3,4]. It is reported that hyperopia increases or remains stable until 5 to 10 years of age and then decreases with age [5,6,7]. In most cases, AccET is accompanied by hyperopia and astigmatism, and it is initially treated by wearing glasses for full refractive error correction. However, even after hyperopic refractive errors have been corrected, there are some cases where decompensated AccET or visual development disorders such as amblyopia occur [8,9]. The amount of hyperopia and the angle of esotropia in children with AccET tend to decrease after spectacle prescription, and as children age, some may discontinue spectacle wear [10,11]. Several studies have observed longitudinal changes in the spherical equivalent refractive error (SE) of children with AccET, but these studies are limited due to absence of control groups and relatively small numbers of patients [9,10,12]. In this study, we compare the long-term changes in refractive errors between hyperopic children with and without AccET, and investigated the effect of amblyopia, initial diopters and age of first spectacles prescription on changes in refractive error.

## 2. Materials and Methods

### 2.1. Participants

This is a retrospective study using medical records of hyperopic patients with an ocular diagnosis of more than 0.75 diopter with or without AccET at initial visits who underwent cycloplegic refraction at Kangbuk Samsung Hospital between January 2000 and January 2017. Exclusion criteria were children with infantile esotropia, partially accommodative esotropia, previous strabismus surgery, congenital malformations or neurologic diseases, paretic or restrictive strabismus, and sensory esotropia. Amblyopia was defined as an inter-ocular difference in best-corrected visual acuity of 2 or more logarithm of the minimal angle of resolution (logMAR) lines. The SE was calculated as the sum of the sphere plus one half of a cylinder. Cylinder powers were recorded using minus cylinder notation. Anisometropia was defined as an inter-ocular difference in power of 2 or more diopters. All patients were followed up for a minimum of 3 years after spectacle prescription and had a follow-up interval of less than a year. We followed all related tenets of the Declaration of Helsinki, and this work was approved by the Institutional Review Board of Kangbuk Samsung Hospital in Seoul, Korea. Informed consent was waived because this study was retrospective and had minimal risk of harm to participants.

Participants were classified into two groups: Those who with hyperopia and no AccET were assigned to group 1, and those with AccET were assigned to group 2. All patients underwent full ophthalmologic examination including best-corrected visual acuity; cycloplegic refraction; fundus examination; angles of deviation, which were measured by simultaneous and alternate prism cover tests; ductions and versions; and stereopsis, which was assessed by the Titmus test. Good stereopsis was defined as less than 100 s of arcs. Angle of deviation, Bagolini striated glasses, and Worth-4-dot tests were evaluated at distance (6 m) and near (33 cm) and were performed to evaluate binocularity. Axial lengths were obtained with IOLMaster 500 (Carl Zeiss Meditec, Oberkochen, Germany). Refractions were performed using retinoscopy after the instillation of 1% cyclopentolate and 0.5% tropicamide eyedrops three times within 5-min intervals by the pediatric ophthalmologist as a part of the routine ophthalmic examination.

Spectacles were prescribed with full correction based on cycloplegic refraction at initial visit. In the case where visual acuity in both eyes was better than logMAR 0.0 while maintaining orthotropic alignment after wearing prescribed glasses, spectacles were prescribed by gradual reduction of hyperopic diopter with annual cycloplegic refraction. When the esotropia recurred or persisted, cycloplegic refraction was re-performed to determine whether there was latent hyperopia. 

### 2.2. Data Analysis and Statistics

Data analysis was conducted using SPSS Statistics (Version 24.0, SPSS Inc., Chicago, IL, USA) software. Continuous data were compared using independent sample *t*-test and represented as mean ± standard deviation (SD). Categorical variables are shown as ratios and were compared with the Pearson Chi-Square test or Fisher’s exact test. Linear mixed models were applied to analyze of repeated postoperative angle deviation at different time periods. 

The longitudinal changes of SE, which were repeatedly evaluated according to the time after spectacles prescribed, were analyzed using a general linear mixed model [13]. The interaction between groups and time was also calculated using general linear mixed model analysis with considering missing value due to decreasing the number of patients who followed up after 3 years. The linear mixed models were conducted using Proc Mixed from the Statistical Analysis System (SAS institute, Inc., Carey, NC, USA).

## 3. Results

A total of 456 patients met the inclusion criteria for this study: 190 (41.7%) in the hyperopic control group (group 1) and 266 (58.3%) in the AccET group (group 2). The clinical baseline characteristics of patients in both groups are shown in Table 1. The mean age of the first visit to the hospital was 7.7 ± 3.2 in group 1 and 8.1 ± 4.2 in group 2. The mean esodeviation at initial visit in group 2 was 18.85 ± 11.63 PD at distance and 24.37 ± 12.79 PD at near, which decreased after correction with spectacles to 4.00 ± 7.18 PD at distance and 6.88 ± 8.05 PD at near. All patients attended annual follow-up visits for at least 3 years and had routine ophthalmic examinations. The mean follow-up period was 5.46 ± 3.08 (3.33–10.25) years in group 1 and 8.08 ± 3.25 (3.08–17.17) years in group 2. The mean follow-up interval was 5.78 ± 3.93 months in group 1 and 6.03 ± 3.39 months in group 2. The number of patients according to follow-up period was illustrated in the supplementary material Table S1.

**Figure 1:**
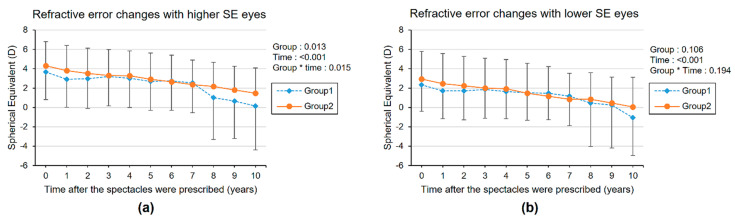
The longitudinal changes of mean spherical equivalent refractive error (SE) of eyes with higher SE values (**a**) and lower SE values (**b**) at initial spectacle prescription in total hyperopic children. The group × time interaction effect was analyzed by linear mixed model analysis. Group 1 = patients with hyperopia without refractive accommodative esotropia. Group 2 = patients with refractive accommodative esotropia.

**Figure 2:**
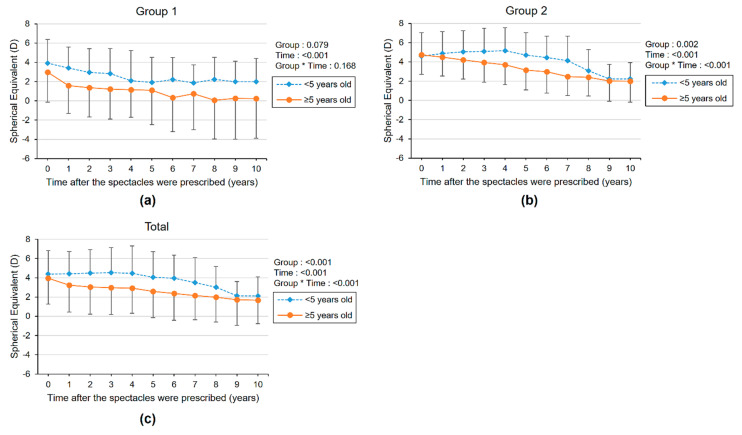
The longitudinal changes of mean spherical equivalent refractive error (SE) with higher SE eyes after spectacle prescription for children in the hyperopic control group (group 1) (**a**), refractive accommodative esotropia group (group 2) (**b**), and total hyperopic children (**c**). The subgroup was divided according to the age of first prescribed spectacles (<5 years old versus ≥5 years old). The group × time interaction effect was analyzed by linear mixed model analysis.

We evaluated the longitudinal refractive error changes of eyes with higher SE and lower SE after spectacle prescription. (Figure 1, Table S2) The mean initial SEs of eyes with higher SE values were 3.66 ± 2.84 and 4.31 ± 2.48 in groups 1 and 2, respectively, and the mean initial SEs of eyes with lower SE values were 2.34 ± 2.73 and 2.93 ± 2.85 in groups 1 and 2, respectively. Both groups 1 and 2 showed a decreasing tendency in SE during the follow-up period, and the children in group 1 showed a greater decrease in SE over time compared with the children in group 2; this tendency was significantly different between groups in eyes with higher SE (group × time *p*-value = 0.015). All amblyopic eyes were eyes with higher SE. There were no significant changes in lower SE refractive eye with linear mixed model (group × time *p*-value: 0.194). Therefore, we compared the longitudinal changes of SE with eye for subgroups with higher SE values. The mean SE of eyes with higher SE values gradually decreased after spectacle prescription for all ages in group 1. In group 2, SE increased for first 4 years after spectacle prescription and then continued to decrease thereafter. Subgroup analysis was performed according to the age of first prescribed corrective lenses (<5 years old versus ≥5 years old). (Figure 2, Table S3) The mean SE gradually decreased after spectacle prescription for all ages in group 1 and for children who were prescribed spectacles after the age of 5 in group 2. Exceptionally, in group 2, of children under 5 years of age when starting initial spectacle use, the SE increased during the first 5 years after using corrective lenses and then decreased thereafter. 

**Figure 3:**
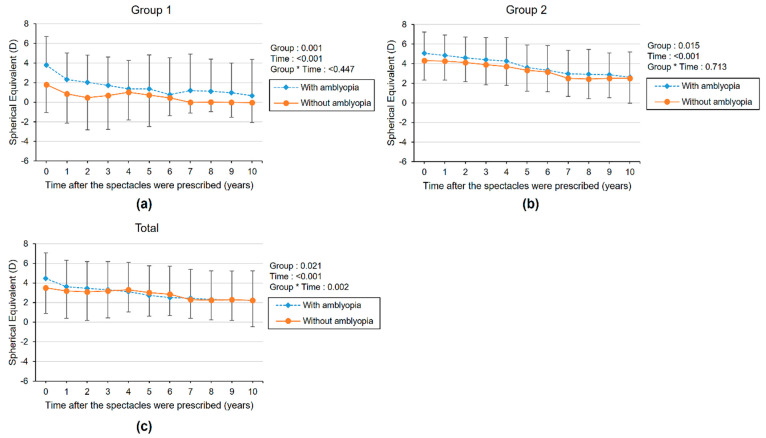
The longitudinal changes of mean spherical equivalent refractive error with higher spherical equivalent eyes after spectacle prescription for children according to the presence of amblyopia in the hyperopic control group (group 1) (**a**), refractive accommodative esotropia group (group 2) (**b**), and total hyperopic children (**c**). The group × time interaction effect was analyzed by linear mixed model analysis.

**Figure 4:**
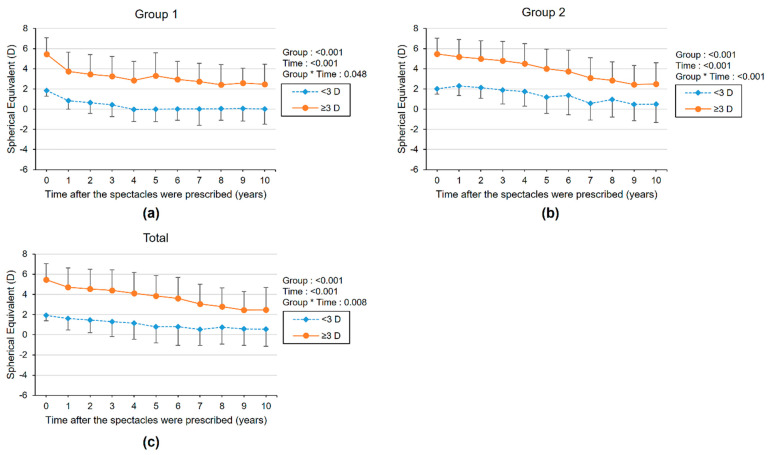
The longitudinal changes of mean spherical equivalent refractive error (SE) with higher SE eyes after spectacle prescription for children in hyperopic control group (group 1) (**a**), refractive accommodative esotropia group (group 2) (**b**), and total hyperopic children (**c**). Subgroups were divided by initial SE of <3.0 D and ≥3.0 D. The group × time interaction effect was analyzed by linear mixed model analysis.

Furthermore, we compared the longitudinal changes in SE according to the presence of amblyopia (Figure 3, Table S4). SE gradually decreased regardless of amblyopia, but the initial SE was higher in the amblyopia subgroup. Reduction of SE was greater in the amblyopia subgroup than in the non-amblyopia subgroup for total subjects, but the difference was not statistically significant (*p* = 0.07). Children were also divided into two subgroups according to the initial degree of SE (Figure 4, Table S5). The SE showed a significantly greater decrease in the more hyperopia (≥3 D) subgroup than in the less hyperopia (<3 D) subgroup. (Group × time *p*-value = 0.048 and <0.001 in groups 1 and 2, respectively).

## 4. Discussion

AccET is a type of strabismus that is caused by accommodative convergence associated with hyperopia, and most children with AccET can improve their binocular vision with long-term proper treatment [14,15]. Therefore, examination of refractive error in patients with AccET is important not only for the initial visit but also for follow-up observations. In the present study, we investigated the longitudinal changes in the SE values after spectacle prescription over time in children with hyperopia with or without AccET. The mean SE gradually decreased after spectacle prescription for all ages in children in the hyperopic control group. However, the mean SE increased and peaked at the first 4 years after spectacle prescription and then progressively decreased in children with AccET who were less than 5 years old at the time of initial spectacle use. When comparing changes of SE according to initial SE values, larger initial SE values correlated with larger annual SE decreases. 

Several studies have investigated longitudinal changes of SE in children with AccET and reported diverse results. Berk et al. reported that the mean SE decreased by an average of 0.15 D/year at the 3-year follow-up and 0.16 D/year at the 5-year of follow-up regardless of age [11]. Raab et al. reported that the SE gradually increased until patients were 7 years old (0.19 D/year) and then decreased thereafter in children with AccET [4,7]. Additionally, they reported that between ages 7 and 13 years old the general childhood population (0.22 D/year) showed larger reduction of hyperopia than was seen in children with AccET (0.18 D/year). In our study, an initial increase in SE was only observed in children under the age of five following the first spectacle prescription with AccET (a 0.15 D/year increase for the first five years and then decreased to 0.52 D/year, overall, 0.34 D/year decrease), while the SE of other subgroups steadily decreased after wearing corrective lenses. The decrease in hyperopic diopter was greater in the general hyperopic population (−0.56 D/year), which is consistent with Raab et al.’s results. However, the amount of reduction of hyperopic diopter was larger than that seen by Rabb et al. and Berk et al., probably due to ethnic differences. Interestingly, 80% of East Asian young adults and children have a higher prevalence of myopia and a higher progression rate of myopia [16,17,18]. Intense near-sighted work coupled with the highly competitive education system and limited outdoor activities in East Asian children may be involved in an induced myopic shift, and similar environmental factors may influence a greater reduction of hyperopia toward emmetropization. The higher initial SE demonstrated a faster annual decrease, which was similar to previous studies that reported that the degree of emmetropization proceeds according to the initial hyperopia diopter [19,20].

Newborn humans usually have a hyperopic refractive error in the range of +1.00 D to +2.50 D, and fewer than 25% of infants have myopic eyes [21,22,23,24]. During their first year of life, the eyes of low hyperopic infant generally undergo a process of emmetropization [25,26,27] with ocular growth. Several studies showed that wearing plus power lenses especially at an early age is one factor interfering with emmetropization [26,28,29]. Lambert et al. and Biler et al. have reported that children with AccET are less likely to undergo the emmetropization process [30,31]. Similarly, this study also showed that initial SE increased and prolonged emmetropization in younger AccET children, which was different than what was seen in the hyperopic control group or older children with AccET. These results are consistent with the result of Lambert and Lynn, which evaluated the longitudinal changes in the SE of children with AccET based on age at the time spectacles were prescribed and reported that the SE relatively decreased slower over time when the age of the initial spectacle correction was younger [10]. They suggested that the age of initial use of spectacles affects changes in refractive error as the eyes of fast-growing younger children may be sensitive to changes in environmental factors [10]. Although the reason is not clear, hyperopic correction and the intrinsic factor of AccET itself may affect the process of emmetropization in children with AccET. One intrinsic cause of interfering emmetropization may be the abnormal binocular vision of children with AccET. Children with AccET often suffer abnormal binocular vision, and one third of them have amblyopia [32,33]. The prevalence of anisometropia, a risk factor for amblyopia development in children with AccET, was 20 to 40%, which is 10-fold higher than that in normal children [34,35]. Park et al. reported that amblyopia and abnormal binocular vision are among the most important factors that interrupt ocular growth regulation [12]. However, there was no significant difference of amblyopia risk or good stereopsis in either group in this study; this is probably because all subjects were patients in tertiary hospital, and the hyperopic control group tended to have anisometropia or amblyopia for treatment. Thus, future research is needed to compare the changes of SE with AccET and the general hyperopic population.

In this study, we also compared the longitudinal changes in the SE according to the presence of amblyopia. Longitudinal reduction in the SE was greater in the amblyopic subgroup than in the non-amblyopic subgroup of the total population. Similar to our study, some studies have reported that hyperopic correction in amblyopic eyes induced a greater reduction in hyperopic diopter than in non-amblyopic eyes [12,36]. The blurred images on the retina caused by amblyopia might affect the changes in refractive error. If the clear images were focused on the retina due to hyperopic correction in children with hyperopia, the myopic shift stimulation toward emmetropia could be removed, which may delay the process of emmetropization. However, if children with amblyopia cannot obtain clear corrected vision even with corrective lenses, it could be postulated that hyperopic correction rarely interferes with the process of emmetropization in amblyopic eyes. This result also suggests that non-amblyopic eyes might be more sensitive to spectacle correction on emmetropization than amblyopic eyes. 

There were several limitations in our study. First, there was a slight difference in the number of children in the two groups, and the number of patients in hyperopic control group was halved after 5 years of follow-up likely because children with only simple hyperopia without any complications are unlikely to visit tertiary hospital or do not follow-up continuously. Furthermore, hyperopic control children without accommodative esotropia in this study do not represent the general normal hyperopic population because they were also patients who visited the tertiary hospital and tended to have anisometropia or amblyopia. Second, a control group of age-matched patients of hyperopia with or without AccET who did not wear glasses is needed for accurate comparison to reveal the effects of spectacles on the change in refractive error. However, this was retrospective study, and it is unethical to not prescribe spectacles when needed, especially for AccET patients. Prospective study including the general hyperopic population are needed in the future.

The pattern of decrease of SE in AccET children was different from hyperopic children without esotropia. In the present study, SE of hyperopic children decreased in a time dependent manner regardless of the presence of AccET, though the course was different in younger AccET patients. Although wearing corrective lenses is the treatment of choice in AccET, it is possible that long-term spectacle wearing could affect emmetropization. Emmetropization was faster in hyperopic patients without AccET than in those with AccET. Initial hyperopia was higher in younger patients and those who had amblyopia. Further research is needed to determine the exact mechanism of hyperopic correction on emmetropization and to minimize its effects.

## Figures and Tables

**Table 1:** Baseline characteristics of hyperopic patients with or without refractive accommodative esotropia.

Variables	Group 1 (*n* = 190)	Group 2 (*n* = 266)	*p*-Value
Age at initial spectacle use (years old)	7.7 ± 3.2	8.1 ± 4.2	0.212 ^1^
Sex			0.601 ^2^
Male	96 (50.53)	141 (53.01)	
Female	94 (49.47)	125 (46.99)	
Axial length (mm) in more hyperopic eye	22.79 ± 1.57	21.98 ± 1.19	0.001 ^1^
Cycloplegic refraction (spherical equivalent) (diopter)	3.11 ± 3.04	4.70 ± 2.11	0.001 ^1^
Baseline esodeviation angle without spectacle (PD)			
Near	1.52 ± 2.87	24.37 ± 12.79	<0.001 ^1^
Distance	0.54 ± 1.63	18.85 ± 11.63	<0.001 ^1^
Baseline esodeviation angle with spectacle (PD)			
Near	0.73 ± 2.24	6.88 ± 8.05	<0.001 ^1^
Distance	0.43 ± 1.53	4.00 ± 7.18	<0.001 ^1^
Amblyopia	102 (58.95)	132 (49.62)	0.057
Anisometropia	42 (22.11)	69 (25.94)	0.347
Good stereopsis (<100 arc seconds)	117 ± 101.51	131 ± 116.69	0.183
Worth-4-dot test fusion	187 (98.42)	260 (97.74)	>0.999 ^2^
Bagolini Striated Glasses Test fusion	179 (98.35)	259 (97.37)	>0.999 ^2^
Follow-up period (years)	5.46 ± 3.08	8.08 ± 3.25	<0.001 ^1^

Group 1 = patients with hyperopia without refractive accommodative esotropia. Group 2 = patients with refractive accommodative esotropia. Data are presented as mean ± standard deviation or n (%). PD, prism diopters. ^1^ Independent *t*-test. ^2^ Pearson Chi-Square test.

## Data Availability

All data generated or analyzed during this study are included in this published article.

## Supplementary Materials

The following are available online at https://www.mdpi.com/article/10.3390/diagnostics11091547/s1.
