# Supplementary material for: Comparison of Longitudinal Changes in Refractive Error of Hyperopic Children with or without Refractive Accommodative Esotropia
Source: Diagnostics (Basel). 2021 Aug 26;11(9):1547. doi: 10.3390/diagnostics11091547 (PMC8466867; doi:10.3390/diagnostics11091547)
Supplement: Supplementary file 1 [file diagnostics-11-01547-s001.zip › diagnostics-1300222-supplementary.pdf]

Supplementary Materials

Table S1 : The number of patients with or without accommodative esotropia according to follow up period. Group 1 = patients with hyperopia without refractive accommodative esotropia. Group 2 = patients with refractive accommodative esotropia.

Follow up period (years)	Group 1 (N)	Group 2 (N)
0	190	266
1	190	266
2	190	266
3	190	266
4	188	245
5	167	217
6	151	197
7	133	181
8	80	160
9	52	139
10	25	109

Table S2 : Data of the longitudinal changes of mean spherical equivalent refractive error (SE) of eyes with higher SE values (a) and lower SE values (b) from initial spectacles prescription to 10 years of follow up. Group 1 = patients with hyperopia without refractive accommodative esotropia. Group 2 = patients with refractive accommodative esotropia.

(a)

Follow up period (years)	SE (diopters)	
	Group 1	Group 2
0	3.66±2.84	4.31±2.48
1	2.92±2.90	3.81±2.60
2	2.97±3.07	3.51±2.62
3	3.21±3.05	3.29±2.69
4	3.02±3.04	3.27±2.58
5	2.70±3.00	2.91±2.70
6	2.75±3.04	2.66±2.76
7	2.53±3.08	2.36±2.53
8	1.03±4.33	2.17±2.48
9	0.65±3.87	1.82±2.43
10	0.14±4.52	1.47±2.60

(b)

Follow up period (years)	SE (diopters)	
	Group 1	Group 2
0	2.34±2.73	2.93±2.85
1	1.72±2.88	2.46±3.11
2	1.71±2.99	2.23±3.06
3	1.85±2.95	2.01±3.09
4	1.64±2.81	1.94±3.01

5	1.52±2.86	1.48±3.08
6	1.46±2.71	1.17±3.05
7	1.17±3.05	0.86±2.66
8	0.45±4.47	0.84±2.76
9	0.25±4.44	0.45±2.68
10	-1.06±3.89	0.05±3.07

Table S3 : Data of the longitudinal changes of mean spherical equivalent refractive error (SE) of eyes with higher SE values from initial spectacles prescription to 10 years of follow up for children in the hyperopic control group (group 1) (a), refractive accommodative esotropia group (group 2) (b), and total hyperopic children (c). The subgroup was divided according to the age of first prescribed spectacles (< 5 years old versus ≥5 years old). Group 1 = patients with hyperopia without refractive accommodative esotropia. Group 2 = patients with refractive accommodative esotropia.

(a)

Follow up period (years)	SE (diopters)	
	Initial age < 5 years old	Initial age ≥5 years old
0	3.92±2.45	2.97±3.11
1	3.42±2.17	1.58±2.90
2	2.95±2.46	1.36±3.03
3	2.84±2.60	1.21±3.10
4	2.09±3.13	1.16±2.85
5	1.93±2.61	1.10±3.57
6	2.21±2.29	0.33±3.52
7	1.87±1.87	0.74±3.73
8	2.22±2.32	0.06±4.03
9	2.00±2.11	0.25±4.24
10	1.99±2.41	0.23±4.12

(b)

Follow up period (years)	SE (diopters)	
	Initial age < 5 years old	Initial age ≥5 years old
0	4.59±2.44	4.72±2.02
1	4.87±2.27	4.48±1.96
2	5.04±2.21	4.20±1.98
3	5.09±2.39	3.93±2.04
4	5.16±2.39	3.70±2.05
5	4.71±2.33	3.15±2.06
6	4.45±2.22	2.97±2.22
7	4.14±2.52	2.48±1.98
8	3.07±2.20	2.41±1.95
9	2.23±1.50	2.02±2.11
10	2.22±1.71	1.98±2.16

(c)

Follow up period (years)	SE (diopters)	
	Initial age < 5 years old	Initial age ≥5 years old
0	4.39±2.45	3.96±2.69
1	4.41±2.33	3.23±2.80
2	4.48±2.45	3.05±2.83
3	4.54±2.61	2.97±2.78
4	4.47±2.85	2.92±2.60
5	4.06±2.65	2.58±2.72
6	3.96±2.39	2.37±2.79
7	3.50±2.60	2.15±2.51
8	3.01±2.16	1.99±2.59
9	2.13±1.47	1.72±2.66
10	2.11±1.98	1.68±2.44

Table S4 : Data of the longitudinal changes of mean spherical equivalent refractive error (SE) of eyes with higher SE values from initial spectacles prescription to 10 years of follow up for children according to the presence of amblyopia in the hyperopic control group (group 1) (a), refractive accommodative esotropia group (group 2) (b), and total hyperopic children (c). Group 1 = patients with hyperopia without refractive accommodative esotropia. Group 2 = patients with refractive accommodative esotropia.

(a)

Follow up period (years)	SE (diopters)	
	With Amblyopia	Without Amblyopia
0	3.79±2.91	1.80±2.86
1	2.31±2.71	0.84±2.98
2	2.04±2.76	0.46±3.28
3	1.71±2.90	0.68±3.44
4	1.36±2.92	1.02±2.83
5	1.35±3.49	0.72±3.20
6	0.79±3.76	0.45±1.82
7	1.18±3.72	-0.02±1.10
8	1.11±3.30	0.01±0.97
9	0.98±3.02	-0.02±1.51
10	0.66±3.71	-0.05±2.02

(b)

Follow up period (years)	SE (diopters)	
	With Amblyopia	Without Amblyopia
0	5.06±2.17	4.32±1.99
1	4.83±2.09	4.27±1.94

2	4.61±2.13	4.12±1.96
3	4.42±2.24	3.90±2.05
4	4.26±2.41	3.71±1.92
5	3.62±2.28	3.32±2.12
6	3.33±2.54	3.17±2.03
7	2.96±2.40	2.50±1.82
8	2.91±2.55	2.44±2.01
9	2.86±2.23	2.51±1.99
10	2.62±2.56	2.50±2.54

(c)

Follow up period (years)	SE (diopters)	
	With Amblyopia	Without Amblyopia
0	4.45±2.63	3.49±2.59
1	3.62±2.71	3.19±2.81
2	3.44±2.75	3.10±2.90
3	3.31±2.86	3.19±2.76
4	3.12±2.98	3.31±2.27
5	2.74±3.01	3.02±2.39
6	2.52±3.20	2.85±2.18
7	2.43±2.95	2.29±1.90
8	2.31±2.94	2.25±2.02
9	2.28±2.95	2.30±2.10
10	2.23±3.01	2.22±2.68

Table S5 : Data of the longitudinal changes of mean spherical equivalent refractive error (SE) of eyes with higher SE values from initial spectacles prescription to 10 years of follow up for children in the hyperopic control group (group 1) (a), refractive accommodative esotropia group (group 2) (b), and total hyperopic children (c). The subgroup was divided by initial SE of <3.0 D and ≥3.0 D. Group 1 = patients with hyperopia without refractive accommodative esotropia. Group 2 = patients with refractive accommodative esotropia.

(a)

Follow up period (years)	SE (diopters)	
	Initial SE <3.0 D	Initial SE ≥3.0 D
0	1.84±0.57	5.45±1.63
1	0.83±0.83	3.73±1.92
2	0.63±1.05	3.46±1.96
3	0.42±1.17	3.25±1.97
4	-0.04±1.18	2.84±1.89
5	-0.02±1.24	3.31±2.27
6	0.02±1.12	2.94±1.80
7	0.02±1.62	2.72±1.82

8	0.04±1.15	2.41±2.01
9	0.06±1.24	2.58±1.48
10	0.02±1.51	2.46±2.00

(b)

Follow up period (years)	SE (diopters)	
	Initial SE <3.0 D	Initial SE ≥3.0 D
0	2.00±0.51	5.47±1.58
1	2.29±0.95	5.19±1.71
2	2.12±1.03	5.00±1.78
3	1.89±1.39	4.79±1.91
4	1.74±1.45	4.52±1.97
5	1.20±1.62	4.00±1.93
6	1.37±1.92	3.73±2.11
7	0.57±1.65	3.10±1.99
8	0.94±1.73	2.85±1.84
9	0.46±1.62	2.44±1.90
10	0.50±1.83	2.50±2.10

(c)

Follow up period (years)	SE (diopters)	
	Initial SE <3.0 D	Initial SE ≥3.0 D
0	1.92±0.54	5.46±1.60
1	1.61±1.15	4.72±1.90
2	1.46±1.27	4.54±1.96
3	1.30±1.49	4.39±2.04
4	1.14±1.60	4.11±2.07
5	0.80±1.60	3.85±2.02
6	0.80±1.85	3.60±2.08
7	0.52±1.60	3.04±1.96
8	0.74±1.67	2.79±1.86
9	0.57±1.61	2.46±1.84
10	0.56±1.70	2.48±2.22
